# Assessment of the Psychometric Properties of the Holland Sleep Disorders Questionnaire in the Iranian Population

**DOI:** 10.1155/2022/1367067

**Published:** 2022-03-14

**Authors:** Habibolah Khazaie, Amir Jalali, Reza Mohammadi, Azita Chehri, Amirhossein Khazaie

**Affiliations:** ^1^Sleep Disorders Research Center, Kermanshah University of Medical Sciences (KUMS), Kermanshah, Iran; ^2^Substance Abuse Prevention Research Center, Research Institute for Health, Kermanshah University of Medical Sciences, Iran; ^3^Department of Psychology, Kermanshah Branch, Islamic Azad University, Kermanshah, Iran

## Abstract

**Background:**

Assessing sleep disorders and understanding their causes are essential for the proper treatment and management of the disorders. The Holland Sleep Disorders Questionnaire (HSDQ) is a self-assessment questionnaire that measures sleep problems and symptoms based on the six categories of sleep disorders described in the International Classification of Sleep Disorders-2 (ICSD-2). The aim of this study was at validating and assessing the psychometric properties of the HSDQ in Iranian adults.

**Method:**

The study was carried out as a methodological and validation work. The guidelines for translation and cultural adaptation of patient-reported outcome measures were followed for the translation and the cultural validation of the tool. To examine construct validity, exploratory factor analysis (EFA) with 216 participants and confirmatory factor analysis (CFA) with 355 participants were used. As to the reliability, the test-retest method and, as to internal consistency, Cronbach's alpha were employed. Data analyses were done in SPSS-25 and LISREL-8.

**Results:**

The CFA and EFA results confirmed the tool with six factors and 31 items. The *R*^2^ index of the model was 0.99, which indicated that 99% of changes in the dependent variable (adults' sleep problem) were attributed to the independent variable (the 31 items). In other words, 0.99 of the changes in the dependent variable were due to the independent variables. The main indices of CFA (*χ*^2^/DF = 2.65, CFI = 0.91NNFI/TLI = 0.92GFI = 0.81, REMSEA = 0.043, *R*^2^ = 0.99) were acceptable. In addition, a correlation coefficient below 0.05 was considered as significant. Reliability of the tool based on internal correlation (Cronbach's alpha) was in the 0.701–0.924 range for the subscales and equal to 0.789 for the whole tool.

**Conclusion:**

In general, the results showed that the Farsi version of HSDQ (six factors and 31 items) had acceptable and applicable indices and it can be used as a valid tool in the Iranian society. The tool can be used as a reliable tool in different fields of medical sciences.

## 1. Introduction

Sleep is a process that is essential for maintaining brain function, and the lack of it can lead to memory and attention impairments [1]. Sleep problems are hazardous for health and treating them is very expensive [2]. Sleep disorders are comorbid of other diseases such as an increased risk of obesity, diabetes, hypertension, tachycardia, and stroke [3]. Sleep disruptions have short-term health consequences (somatic pain, emotional distress, mood disorders, and cognitive, memory, and performance deficits) and also long-term health consequences (hypertension, dyslipidemia, cardiovascular disease, weight-related issues, and colorectal cancer) [4].

Through decreasing the quality of life, sleep deprivation imposes risks to physical, mental, social, and emotional health. In addition, low sleep quality has a relationship with an increase in stress, depression, irritation, and lower satisfaction with life [5]. Changes in sleep pattern are one of the earliest behavioral symptoms of psychophysical problems [6] such as somatic pain, emotional distress, and weight-related issues [4]. The lack of balance in sleep and rest process can create excessive fatigue and nervousness [7].

Studies based on questionnaires have shown the high prevalence of sleep disorders in many countries [8–12]. All around the world, these disorders cause negative effects on one's quality of life [12]. Epidemiological studies have shown that about 36% of the total adult United State (US) citizens sleep less than seven hours overnight [13]. A multinational study reported that the prevalence of sleep problems in the United State of America (USA) was the highest (56%), followed by European countries (23–26%) and Japan (23%) [14].

There is a fast-growing number of studies on sleep epidemiology [8, 10, 12], which is due to the gradual increase in public awareness about the negative effects of inadequate and irregular sleep on human error and health [15]. Therefore, there is a need for collecting correct and reliable data about the prevalence of sleep disorders and also answering questions about epidemiological data and general health [10].

In general, sleep disorders are assessed using polysomnography, actigraphy, and questionnaires (e.g., Pittsburgh Sleep Quality Index, Sleep Hygiene Index, and Insomnia Severity Index) [16–18]. Holland Sleep Disorders Questionnaire (HSDQ) is a credible questionnaire based on the International Classification of Sleep Disorders-2 (ICSD-2) [19]. The questionnaire measures sleep problems of patients in six main categories of sleep disorders. These six categories are insomnia, Circadian rhythm sleep disorders (CRSD), parasomnia, hypersomnia, restless legs syndrome (RLS)/periodic limb movement disorder (PLMD), and sleep-disordered breathing (SDB). Using the scale, a physician can evaluate which one of these sleep disorders has inflicted the patient [10, 20].

Given the introduction about the problems, costs, and high prevalence of sleep disorders, one of the ways to collect useful information to attenuate and treat the disorders is epidemiological study. There is no valid and integrated tool for Iranian populations to measure sleep disorders. By searching for specific researches and texts related to sleep, we were convinced that there was no suitable tool to assess sleep problems in different categories as insomnia, CRSD, parasomnia, hypersomnia, RLS/PLMD, and SDB. Although the HSDQ is not based on an updated version of the classification of sleep disorders (ICSD-2), considering that the items match the Iranian culture and the classification of sleep disorders into six categories, it was used in this study, and along with cultural validation, the psychometric properties of the HSDQ in Iranian adult were also evaluated. Taking into account that HSDQ has proper items and categorizations and it is a simple and inexpensive tool to use, it can be a good option for the Iranian population. Therefore, the present study is an attempt to examine psychometric properties of HSDQ in the Iranian population.

## 2. Methods

### 2.1. Setting

The study was carried out as a methodological and validation work.

### 2.2. Participants

The study population consisted of adults in age range of 18–85 living in Kermanshah city. The characteristics of the participants are presented in Table 1.

To perform cluster sampling, six urban districts were selected among 10 urban districts of the city. Then, three districts were randomly selected and six clinics (private or public) were selected randomly from these three districts. The participants were selected based on a set of inclusion criteria among the persons who had a sleep disorder file in the clinics (*n* = 230). Inclusion criteria were residence in Kermanshah city, reading and writing literacy, interested in participation, no dependence on psychedelic and narcotic drugs, and no physical and psychological problems in the last six months, except for sleep disorders. In addition, 300 individuals without sleep problem who visited the clinics for skin and hair problems were selected.

The sample size for face validity included 10 eligible adult persons (with a sleep disorder file) in Kermanshah city, 12 university professors for the construct validity phase, 216 individuals for exploratory factor analysis (EFA), and 355 individuals for confirmatory factor analysis (CFA) phases (a large number of the questionnaires were not returned or returned not completely filled out).

Given the limitations of the COVID-19 pandemic, the questionnaires were filled out through a blended method. So that hard copies were provided to the individual who had an easy access to the clinics (for EFA, 118 electronic and 112 hard copies were sent, and for CFA, 204 electronic and 151 hard copies were sent) and for others, an electronic version of the tool was sent to them via email or using WhatsApp (an electronic questionnaire link was sent). In this study, an electronic questionnaire was sent to 540 people (86 people via email and 454 people via WhatsApp), out of which only 248 returned and only 204 were usable.

### 2.3. Study Tools

Data gathering tools were a demographics form and HSDQ to measure sleep problems and symptoms including the six main categories of sleep disorders. The tool under study is a credible tool based on ICSD-2 that measures sleep problems and symptoms in six main categories of sleep disorders. The participants were asked to answer the items based on their experience over the past three months. The questionnaire was designed by Kerkhof et al. with 32 items based on Likert's five-point scale (not feasible = 1, usually not feasible = 2, sometimes feasible = 3, usually feasible = 4, and mostly feasible = 5). Kerkhof et al. reported a Cronbach alpha of 0.9, and the coefficients for six factors of sleep disorders ranged from 0.65 (SDB) to 0.78 (CRSD) [20].

### 2.4. Cultural Validity

The guidelines for translation and cultural adaptation of patient-reported outcome measures were followed for the translation and the cultural validation of the tool [21]. The tool was translated independently by two native speakers from English into Farsi. The results were examined by a panel of experts in the presence of the research team members, and one version was developed out of the two translations. Afterwards, the Farsi version of the tool was backward translated into English by two other translators. After revising the translation works, a cultural comparison process was performed. The two translations were compared with the original version to make sure of conceptual equivalence of the two translation works and the original version. Eventually, the final version of the translated tool was sent to the developer of the tool for confirmation. To examine cognitive equivalence, the final translated copy was provided to 10 adult persons with sleep disorder in the sleep disorder clinics in Kermanshah city to examine their ability to comprehend, interpret, and understand the items. The tool was revised based on the cognitive findings to make sure of cultural comparability. Eventually, the final translation was reviewed to spot and remove any grammar error or typos.

### 2.5. Data Analysis

#### 2.5.1. Descriptive Analysis

Demographic variables of research units were examined using relative frequency and content and mean and standard deviation of the mode.

#### 2.5.2. Face Validity

To check face validity, the scale was provided to another 10 adult persons, and in face-to-face interviews, they were asked to highlight any vague item and word or ambiguity or wrong perception in the text.

#### 2.5.3. Content Validity

As to content validity, the scale was provided to 12 researchers, members of faculty boards, and experts in pertinent fields for revising and giving opinions. Through this, content validity was conducted qualitatively [22]. To determine quantitative content validity, the content validity index (CVI) was used based on the Walts and Bassel index [23] on all indices (Table 2).

#### 2.5.4. Construct Validity

In this study, EFA and CFA methods were used to confirm the construct validity. In each stage of EFA and CFA, normal distribution of the data was checked using the multivariate test.

#### 2.5.5. Multivariate Normality Data

The skewness value for each statement varied from −1.09 to 1.86, and it was at a (−2, 2) range. This means that the statements are normal in terms of skewness with symmetric distribution [24]. Moreover, kurtosis ranged from −1.7 to 2.7 (Table 2). Normal distribution of the data in each stage of CFA was checked using skewness and kurtosis. The validity of the model was examined based on the factor load of each item (for *t* value > 1.96, significant level = 95%; and for *t* value > 2.576 & 3.29, significant levels were 99% and 999%, respectively).

#### 2.5.6. Internal Consistency

To examine fitness of the model, the maximum likelihood method was adopted. In addition, to check tool reliability, internal consistency was used using Cronbach's alpha for each item and then for the whole tool.

### 2.6. Ethics Consideration

Along with permission from the developer of the tool, the study was approved by the ethics committee (IR.KUMS.REC.1400.146). The researchers observed research ethics based on the Helsinki declaration.

## 3. Results

### 3.1. Descriptive Results

For EFA, totally, 52.3% of the participants were male and 47.7% were female. The mean age of 216 participants was 39.74 ± 10.41 with minimum and maximum ages equal to 18 and 69 years, respectively. The mean body mass index (BMI) was equal to 27.75 ± 4.91 with min and max BMIs of 18.44 and 41.79, respectively, and the mean of the monthly family income was equal to 280.09 ± 19.42 USD with min and max incomes of 40.35 and 860.96 USD, respectively (Table 1).

For CFA, totally, 61.4% of the participants were male and 38.6% were female. The mean age of 355 participants was 41.92 ± 13.73 with minimum and maximum ages equal to 18 and 85 years, respectively. The mean BMI was equal to 26.43 ± 4.5 with min and max BMIs of 18.12 and 41.79, respectively, and the mean of the monthly family income was equal to 270.14 ± 18.69 USD with min and max incomes of 40.35 and 860.96 USD (Table 1).

### 3.2. Explorative Factor Analysis

EFA was conducted with 216 participants. To make sure of the adequacy of the participants to conduct EFA, the Kaiser-Meyer-Olkin (KMO) test was used (0.741). To examine correlation of the items, Bartlett's test was used (chi-square = 4393.152, degrees of freedom = 796, *p* value = 0.0001). The *p* value for significance of Bartlett's test was less than 0.05. Given the results and significance level, performing EFA on this questionnaire was acceptable [25].

Having checked the requirement for EFA, principal components and varimax rotation were used to extract factors. To determine the number of factors, the three following rules were followed.

1. The factors with Kaiser's criterion (or eigenvalue) are higher than 1 in scree plots and Horn's parallel analysis [26].
2. The factors corresponding to the actual eigenvalue higher than the parallel random eigenvalue were accepted in Horn's parallel analysis. In addition, the factors with actual eigenvalue less than or equal to the mean value of the parallel random eigenvalue were removed as sampling error [27].
3. The items with factor load > 0.3 and higher were loaded on the items under consideration [26].

**Figure 1:**
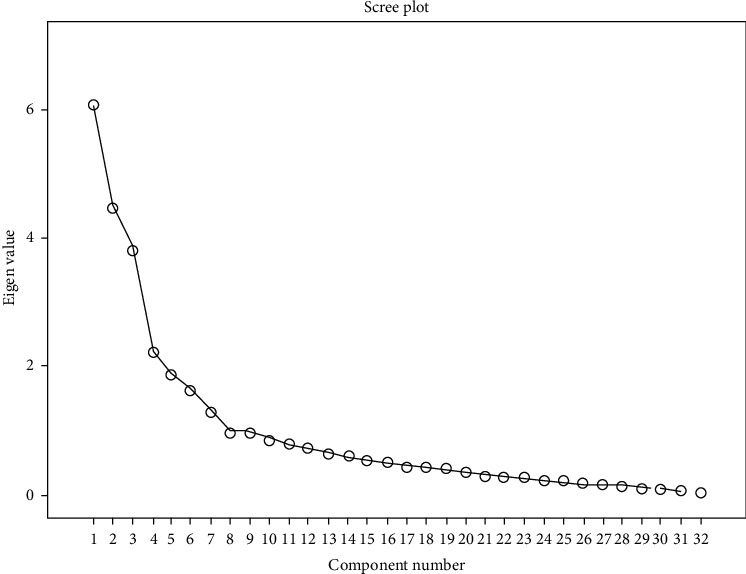
Scree plot of the extractor components of the questionnaire with six fixed factors.

According to these three rules, the primary results showed seven factors with an eigenvalue > 1 for the analysis. In addition, one factor was removed based on Horn's parallel analysis and EFA was repeated with six fix factors (Table 3 and Figure 1).

In general, the results showed (Table 4) that six factors elaborated 62.918% of the variance of 32 items. That is, 14.835% of the variance was attributed to factor 1, 12.509% was attributed to factor 2, 11.466% was attributed to factor 3, 10.8% was attributed to factor 4, 7.624% was attributed to factor 5, and 5.684% was attributed to factor 6. Annexed Table 1 or the rotated factor matrix lists the factor load of each item based on the four factors.

In general, the EFA results confirmed six factor loads [20]. However, the factors' content did not necessary overlap with the constructs of the main article, which was not a necessity condition either. As listed in Annexed Table 1, item no. 14 was loaded on the RLS/PLMD factor, while in Kerkhof et al.'s study [20], it was loaded on insomnia. Eventually, the factors and items were allocated, and to measure internal consistency of the tool, Cronbach's alpha was used (alpha ≥ 0.7 was acceptable, and alpha ≤ 0.5 was unacceptable) [28] (Table 5).

### 3.3. Confirmatory Factor Analysis

**Figure 2:**
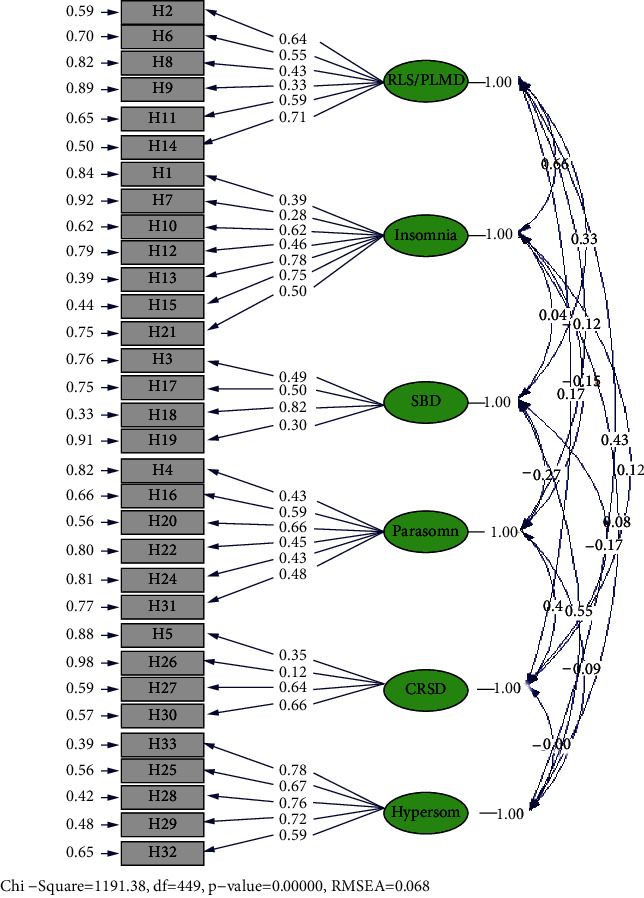
The six-factor model of the HSDQ in the Iranian population standard. CRSD: Circadian rhythm sleep disorders, RLS: restless legs syndrome, PLMD: periodic limb movement disorder; SDB: sleep-disordered breathing; HGDQ: Holland Sleep Disorders Questionnaire.

CFA was carried out with 355 participants on six factors and 32 items. Table 5 and Figure 2 demonstrate CFA in standard condition without coefficient.

As the results showed, none of the items were removed except for item 26 of the CRSD factor as its factor load was less than the critical value (±1.96). In addition, Table 6 lists the goodness of fit indices of CFA. Given the goodness of fit indices listed in Table 6, the model has an acceptable goodness of fit and it fits the collected data.

The reliability of the tool was determined through the test-retest method with participation of 15 participants (not among the main group of participants) who filled out the tool twice with a 10-day interval, and the correlation coefficient was obtained equal to 0.875.

### 3.4. Internal Consistency

To examine internal consistency (internal reliability) of the tool, Cronbach's alpha was obtained for the whole tool with 31 items equal to 0.789. Cronbach's alpha for the subscales of the tool was in the 0.701 and 0.924 range. Therefore, the subscales had the reliability to measure the variables (Table 5).

As listed in Table 7, correlation coefficients between HSDQ and its subscales were positive and significant in all cases. Eventually, based on EFA and CFA, the Farsi version of HSDQ was confirmed for Iranian adults' society with 31 items and six subscales.

## 4. Discussion

The HSDQ was translated into Farsi, and its psychometric properties were examined in the Iranian population. The guidelines for translation and cultural adaptation of patient-reported outcome measures [21] were followed for the translation and the cultural validation of the tool. As to construct validity, EFA was carried out on 216 participants, and afterwards, the number of participants was increased to 355 for CFA.

The EFA results showed that 62.98% of the variance of the 32 items was attributed to the eight factors, and actually, 32 items and six factors were confirmed. Kerkhof et al. analyzed the tool using principal component analysis (PCA) and confirmed it with six factors and 32 items as well [20].

The results showed that item no. 14 was loaded on RLS/PLMD and the factor encompassed six items. However, item 14 in Kerkhof et al.'s questionnaire was placed on the insomnia factor and the RLS/PLMD factor (subscale) had five items [20]. To explain the differences, cultural and traditional differences in the study populations, number of participants, and social condition (e.g., COVID-19 pandemic) that has created severed stresses in Iranian society is notable. At any rate, with a closer look at the item, it is clear that the concept of the item has consistency with the RLS/PLMD factor. Item 14 emphasizes the problems that the person will have in the next day due to ill health and factors such as fatigue, sleepiness, bad mood, poor concentration, memory problems, and lack of energy, in this regard, it can be emphasized that because some of sleep disorders, such as PLMD, occur in deep sleep, the individual is not aware that they have such a disorder. These cases are usually associated with morning symptoms such as drowsiness, fatigue, low mood, and concentration disorders [29]. People with PLMD may also experience sleep deprivation during the day or restless sleep at night [30]. Also, from the concept of item 14, can be understood more to the OSA category, but in the analysis of this study, according to the answers of the participants in the RLS/PLMD category, it can be probable that the lifestyle, culture and attitudes of people towards their situation and problems in the process of responding to the items have been influential in this result.

Based on the CFA results, the item no. 26 in the CRSD factor was removed and the model was finalized with six factors and 31 items with acceptable goodness of fit indices. Kerkhof et al.'s study used PCA in this study [20]. However, in the present study, EFA was used to confirm the validity of the structure, followed by CFA. The CFA is widely used for confirming factors and items [31, 32]. To explain the results, it is notable that the responses of the participants to the items were affected by cultural condition, age, sleep hygiene, life style, and mental condition; therefore, these variables affect the response to all of items in scale.

In addition, reliability of the tool was equal to 0.875 with Cronbach's alpha equal to 0.789 (0.701–0.924). These results confirmed reliability and stability of the tool for the target population. In Kerkhof et al.'s study, Cronbach's alpha was equal to 0.9 (0.73–0.81) [20], which is consistent with the present study.

The correlation coefficient of HSDQ and the subscale were positive and significant in all cases ranging from 0.21 for Parasomnia to 0.75 for RLS/PLMD. The Pearson correlation results in Kerkhof et al.'s study ranged from 0.73 (SDB) to 0.81 (CRSD) [20]. As to the differences between the two studies, differences of the study population and the participants are notable. In Kerkhof et al.'s study [20], the sample size was approximately four times the sample size in the present study. In addition, lifestyle, social interactions, sleep hygiene, monthly income, diet, and even lifestyle are completely different in the two communities studied and these can affect the response to items in the two communities.

Two different groups of participants were selected for EFA and CFA is one of the advantages of this study. In addition, the study design, translation, and cultural validation were based on the ten steps of Wild et al. [21]. Confirmation of the content validity of the tool quantitatively and qualitatively is also among the strengths of this study.

It is notable that the questionnaire is based on ISCD-2 and it is older than ISCD-3. The research team is determined to prepare a revised form of this questionnaire for the Iranian adult community in the future.

## 5. Limitation

Because of the high prevalence of COVID-19 and limitations of finding participants, individuals with and without confirmed sleep disorders were selected. In addition, the HSDQ was administered through the blended method (hard copy and e-form sent via email and WhatsApp account) again because of the limitations caused by the COVID-19 pandemic. The HSDQ represents a screen questionnaire that does not show sleep disorders very accurately. It is also based on the ICSD-2 and is older than the ICSD-3, although the two versions do not differ much in diagnostic classes. And differences in some subclassifications.

## 6. Conclusion

The results indicated that the Farsi version of HSDQ with six factors and 31 items had acceptable indices and applicability for the Iranian society. The tool can be used as a reliable tool in different fields of medical sciences. In general, it can be said that in this study, the HSDQ was validated using appropriate cultural validation methods. Formal validity, content validity quantitatively and qualitatively, structural validity, internal validation, and instrument stability have been performed using standard methods, and finally, this scale has been validated and studied in the Iranian society. Therefore, it can be said that this tool can be used to assess the status of sleep disorders in the Iranian adult community.

## Figures and Tables

**Table 1:** Demographic characteristics of the study participants.

Variable	EFA (216), *n* (%)	CFA (355), *n* (%)
Gender		
Female	103 (47.7)	137 (38.6)
Male	113 (52.3)	218 (61.4)
Marital status		
Unmarried	54 (25)	109 (30.7)
Married	159 (73.6)	242 (68.2)
Divorced & widowed	3 (1.4)	4 (1.1)
Educational level		
Elementary level	36 (16.7)	74 (20.8)
Secondary level	10 (4.6)	38 (10.7)
High school diploma	34 (15.7)	57 (16.1)
Higher education	136 (63)	186 (52.4)
Housing		
Apartment	85 (39.40)	134 (37.7)
Apartment complex	28 (13)	50 (14.1)
Villa house	103 (47.7)	171 (48.2)
Domicile		
City	211 (97.7)	346 (97.5)
Suburb	4 (1.9)	8 (2.3)
Rural area	1 (0.5)	1 (0.3)
Job		
Unemployed	34 (15.7)	49 (13.8)
Employed	122 (56.5)	157 (44.2)
Manual worker	29 (13.4)	59 (16.6)
Freelancer	5 (2.3)	44 (12.4)
Driver	6 (2.8)	19 (5.4)
Student	20 (9.3)	27 (7.6)
Working shift		
Daily	197 (91.2)	320 (90.1)
Night shift	10 (4.6)	16 (4.5)
Circular	9 (4.2)	19 (5.4)
History of sleep disorder		
Yes	73 (33.8)	154 (43.38)
No	143 (66.2)	201 (56.62)
Cigarette smoking		
Yes	12 (5.6)	36 (10.1)
No	204 (94.4)	319 (89.9)
Cigarette smoking line/day		
None	204 (94.4)	319 (89.9)
1–10	4 (1.8)	16 (4.6)
10–20	6 (2.8)	15 (4.2)
20 and more	2 (0.9)	5 (1.5)

**Table 2:** The ratio and index of content validity and multivariate normality index of the tool items.

No.	Items	CVR^a^	CVI^b^	Kurtosis^c^	Skewness^d^
1	During the day, I suffer from fatigue.	0.83	0.92	0.48	−0.7
2	When I lie down in bed, I experience unpleasant, itchy, or burning sensations in my legs.	0.67	0.92	1.65	1.26
3	I wake up with a dry mouth in the morning.	0.67	1	1.47	1.29
4	I wake up in the middle of the night, screaming and/or heavily perspiring and feeling anxious.	0.83	0.92	2.48	1.2
5	I do not fall asleep until the morning and have great difficulty waking up early. I sleep in on weekends.	0.83	1	1.5	1.71
6	When I experience ‘restless legs', I can suppress these sensations by walking or stretching my legs.	0.67	0.83	1.78	1.1
7	The quality of my sleep is poor and I do not feel well rested in the morning.	0.67	0.92	0.38	1.07
8	When I am sitting still, especially in the evenings, I feel an urge to move my legs.	0.50	0.92	1.83	1.68
9	I move my arms or legs during sleep.	0.83	0.83	1.29	1.47
10	At night, I lie awake for a long time.	0.83	0.92	−0.52	0.87
11	While asleep I suffer from kicking leg movements that I just cannot suppress.	0.67	1	−0.13	0.69
12	I worry about the consequences of my poor sleep (e.g., for my health).	0.83	0.83	1.93	1.67
13	I have difficulty falling asleep at night.	0.50	0.92	0.22	0.01
14	Especially after a bad night, I suffer from one or more of the following consequences: fatigue, sleepiness, bad mood, poor concentration, memory problems, and lack of energy.	0.50	0.92	−0.21	0.4
15	Despite having plenty of opportunity to sleep in, I do not get enough sleep.	0.50	0.92	0.006	0.01
16	I regularly have vivid dreams in which I am being attacked and try to defend myself with uncontrolled movements.	0.83	0.92	1.17	0.74
17	I stop breathing during sleep.	0.83	1	−0.17	1.04
18	I snore loudly while I am asleep.	0.67	0.83	−1.07	0.68
19	At night I wake up with a start feeling like I am choking.	0.67	0.92	1.37	1.62
20	I suffer from nightmares or bad dreams.	0.83	0.83	1.67	0.62
21	Because of insufficient sleep, I do not function as well during the day.	0.50	0.92	0.77	1.34
22	I have injured myself during sleep and had no recollection of the event afterwards.	0.83	0.83	1.46	0.32
23	I fall asleep repeatedly throughout the day.	0.50	0.92	1	−1.09
24	Quite often I partially wake up and find myself thrashing my arms. I usually do not recall this later on.	0.50	0.83	2.7	0.89
25	After a daytime nap I do not feel refreshed.	0.67	0.92	2.9	1.86
26	I sleep poorly because I do not manage to fall asleep at a normal hour and wake up at a normal hour in the morning.	0.83	0.92	2.2	1.02
27	When I have to stay awake during the night, my daytime sleep is poor.	0.83	0.83	2.9	1.6
28	I usually sleep more than 10 hours a night, have difficulty waking up in the morning, and nap during the day.	0.67	1	1.3	0.9
29	During the day, I suffer from sleep attacks that are so severe that I cannot suppress them.	0.67	0.92	1.2	1.6
30	The time at which I fall asleep varies strongly from day to day.	0.50	0.92	1.4	0.4
31	I suffer from sleepwalking.	0.50	0.92	2.3	1.9
32	During the day, I fall asleep involuntarily, especially in monotonous situations (e.g., during a boring TV show).	0/83	0.92	2.7	1.9

^a^Content validity ratio: the content validity ratio (Lawshe) is one of the earliest and most widely used methods for quantifying content validity. ^b^Content validity index is the most widely used index in quantitative evaluation. ^c^Skewness is a measure of symmetry or, more precisely, the lack of symmetry. ^d^Kurtosis is a measure of whether the data are heavy-tailed or light-tailed relative to a normal distribution.

**Table 3:** Horn's parallel analysis test results.

Factor	Mean parallel random eigenvalues	Real eigenvalue	Results
1	1.803	6.109	Accept
2	1.688	4.47	Accept
3	1.606	3.84	Accept
4	1.531	2.208	Accept
5	1.47	1.874	Accept
6	1.412	1.632	Accept
7	1.355	1.295	Reject

**Table 4:** Factors extracted after exploratory analysis.

Total variance explained									
Component	Initial eigenvalues			Extraction sums of squared loadings			Rotation sums of squared loadings		
	Total	% of variance	Cumulative %	Total	% of variance	Cumulative %	Total	% of variance	Cumulative %
1	6.109	19.092	19.092	6.109	19.092	19.092	4.747	14.835	14.835
2	4.470	13.970	33.062	4.470	13.970	33.062	4.003	12.509	27.344
3	3.840	11.998	45.061	3.840	11.998	45.061	3.669	11.466	38.810
4	2.208	6.900	51.961	2.208	6.900	51.961	3.456	10.800	49.610
5	1.874	5.856	57.817	1.874	5.856	57.817	2.440	7.624	57.233
6	1.632	5.101	62.918	1.632	5.101	62.918	1.819	5.684	62.918
7	1.295	4.046	66.964						
8	0.998	3.120	70.084						
9	0.973	3.040	73.124						
10	0.880	2.750	75.873						
11	0.776	2.424	78.297						
12	0.730	2.282	80.579						
13	0.664	2.076	82.656						
14	0.613	1.916	84.571						
15	0.549	1.716	86.287						
16	0.527	1.648	87.935						
17	0.457	1.428	89.363						
18	0.429	1.342	90.705						
19	0.405	1.265	91.970						
20	0.385	1.202	93.172						
21	0.332	1.037	94.208						
22	0.302	0.945	95.153						
23	0.258	0.806	95.959						
24	0.234	0.730	96.689						
25	0.230	0.720	97.409						
26	0.184	0.574	97.983						
27	0.154	0.483	98.466						
28	0.145	0.455	98.921						
29	0.121	0.379	99.300						
30	0.093	0.291	99.591						
31	0.081	0.252	99.843						
32	0.050	0.157	100.000						

**Table 5:** *t* value, factor loading correlation, and Cronbach's alpha of the tool items.

Factor	No.	*t* ^a^	*λ* ^b^	Correlation coefficient	Cronbach's alpha	
Insomnia	H1	7.05	0.39^∗∗∗^	0.46	0.8	0.811
	H7	4.81	0.28^∗∗∗^	0.29	0.83	
	H10	11.78	0.62^∗∗∗^	0.61	0.78	
	H12	8.40	0.46^∗∗∗^	0.51	0.79	
	H13	15.91	0.78^∗∗∗^	0.75	0.75	
	H15	15.02	0.75^∗∗∗^	0.72	0.75	
	H21	9.17	0.50^∗∗∗^	0.48	0.798	

Parasomnia	H4	7.10	0.43^∗∗∗^	0.83	0.9	0.924
	H16	10.01	0.59^∗∗∗^	0.68	0.94	
	H20	11.41	0.66^∗∗∗^	0.85	0.897	
	H22	7.54	0.45^∗∗∗^	0.423	0.84	
	H24	7.19	0.43^∗∗∗^	0.81	0.91	
	H31	8.00	0.48^∗∗∗^	0.898	0.895	

CRSD	H5	5.40	0.35^∗^	0.17	0.64	0.701
	H26	1.86	0.12^∗^	0.21	0.25	
	H27	9.17	0.64^∗∗∗^	0.56	0.58	
	H30	9.32	0.66^∗∗∗^	0.51	0.32	

Hypersomnia	H23	16.37	0.78^∗∗∗^	0.75	0.87	0.893
	H25	13.22	0.67^∗∗∗^	0.73	0.87	
	H28	15.82	0.76^∗∗∗^	0.77	0.86	
	H29	14.57	0.72^∗∗∗^	0.82	0.86	
	H32	11.31	0.59^∗∗∗^	0.65	0.89	

RLS/PLMD	H2	12.07	0.64^∗∗∗^	0.61	0.74	0.786
	H6	9.96	0.55^∗∗∗^	0.65	0.73	
	H8	7.54	0.43^∗∗∗^	0.58	0.75	
	H9	5.73	0.33^∗∗∗^	0.34	0.8	
	H11	11.00	0.59^∗∗∗^	0.55	0.75	
	H14	12.67	0.71^∗∗∗^	0.58	0.75	

SBD	H3	8.45	0.49^∗∗∗^	0.46	0.61	0.745
	H17	5.66	0.50^∗∗∗^	0.55	0.55	
	H18	13.7	0.82^∗∗∗^	0.47	0.62	
	H19	5.01	0.30^∗∗∗^	0.41	0.65	

^∗∗∗^ *p* < 0.001; ^∗∗^*p* < 0.01; ^∗^*p* < 0.05. ^a^The calculated values of *t* for all factor loadings of the first and second orders are greater than 1.96 and are therefore significant at the 95% confidence level. ^b^The specific value, which is denoted by the Lambda coefficient and the statistical symbol *λ*, is calculated from the sum of the factors of the factor loads related to all the variables of that factor. CRSD: Circadian rhythm sleep disorders; RLS: restless legs syndrome; PLMD: periodic limb movement disorder; SDB: sleep-disordered breathing.

**Table 6:** Fit indicators confirmatory factor analysis HSDQ.

Fit indicators	Criterion	Level	Interpretation
*χ* ^2^/DF	≤3	2.65	Optimal fit
CFI	<0.9	0.91	Optimal fit
NNFI/TLI	<0.9	0.92	Optimal fit
GFI	<0.8	0.81	Optimal fit
RMSEA	>0.08	0.068	Optimal fit
*R* ^2^	Near 1	0.99	Optimal fit
SRMR	>0.05	0.047	Optimal fit

DF = 449, *p* value = 0.056, chi-square = 1191.38. TLI: Tucker-Lewis index; NFI: normed fit index; GFI: goodness of fit index; RMSEA: root mean square error of approximation; SRMR: standardized root mean square residual; CFI: comparative fit index; *R*^2^: root mean square; *χ*^2^/DF: chi-squared distribution/degrees of freedom.

**Table 7:** Correlation coefficient between HSDQ subscales.

	Parasomnia	Hypersomnia	Insomnia	RLS/PLMD	SBD	CRSD
Parasomnia	1					
Hypersomnia	0.044	1				
Insomnia	0.057	0.101	1			
RLS/PLMD	0.135^∗^	0.381^∗∗^	0.465^∗∗^	1		
SBD	0.036	0.324^∗∗^	0.016	0.211^∗∗^	1	
CRSD	0.170^∗^	0.031	0.285^∗∗^	0.299^∗∗^	0.021	1
HSDQ	0.211^∗∗^	0.681^∗∗^	0.561^∗∗^	0.750^∗∗^	0.613^∗∗^	0.357^∗∗^

^∗^Correlation is significant at the 0.05 level (2 tailed). ^∗∗^Correlation is significant at the 0.01 level (2 tailed). CRSD: Circadian rhythm sleep disorders; RLS: restless legs syndrome; PLMD: periodic limb movement disorder; SDB: sleep-disordered breathing; HSDQ: Holland Sleep Disorders Questionnaire.

## Data Availability

The datasets used in the study are available from the corresponding author upon reasonable request.

## Abbreviations

HSDQ: Holland Sleep Disorders Questionnaire
CVI: Content validity index
CVR: Content validity ratio
KMO: Kaiser-Meyer-Olkin
EFA: Exploratory factor analysis
CFA: Confirmatory factor analysis
TLI: Tucker-Lewis index
NFI: Normed fit index
GFI: Goodness of fit index
CFI: Comparative fit index
R^2^: Root mean square
X^2^/DF: Chi-square distribution/degrees of freedom
RMSEA: Root mean square error of approximation
PCA: Principal component analysis
SRMR: Standardized root mean square residual
KUMS: Kermanshah University of Medical Sciences
CRSD: Circadian rhythm sleep disorders
RLS: Restless legs syndrome
PLMD: Periodic limb movement disorder
SDB: Sleep-disordered breathing
ICSD-2: International Classification of Sleep Disorders-2
BMI: Body mass index.

## Supplementary Materials

Supplementary Materials Annexed Table 1: rotated component matrix.
