# Supplementary material for: Assessment of the Psychometric Properties of the Holland Sleep Disorders Questionnaire in the Iranian Population
Source: Sleep Disord. 2022 Mar 14;2022:1367067. doi: 10.1155/2022/1367067 (PMC8938142; doi:10.1155/2022/1367067)
Supplement: Supplementary Materials — Annexed Table 1: rotated component matrix. [file 1367067.f1.docx]

| **Annexed Table 1. Rotated Component Matrix^a^** | | | | | | | |
| --- | --- | --- | --- | --- | --- | --- | --- |
|  | Component | | | | | | Communalities |
|  | 1 | 2 | 3 | 4 | 5 | 6 | Extraction |
| H1 |  |  | .665 |  |  |  | .520 |
| H2 |  |  |  | .737 |  |  | .636 |
| H3 |  |  |  |  | .682 |  | .490 |
| H4 | .864 |  |  |  |  |  | .761 |
| H5 |  |  |  |  |  | .510 | .345 |
| H6 |  |  |  |  |  |  | .647 |
| H7 |  |  | .430 |  |  |  | .379 |
| H8 |  |  |  | .698 |  |  | .563 |
| H9 |  |  |  | .362 |  |  | .333 |
| H10 |  |  | .651 |  |  |  | .558 |
| H11 |  |  |  | .590 |  |  | .573 |
| H12 |  |  |  |  |  |  | .573 |
| H13 |  |  | .789 |  |  |  | .718 |
| H14 |  |  |  | .532 |  |  | .539 |
| H15 |  |  | .756 |  |  |  | .736 |
| H16 | .790 |  |  |  |  |  | .651 |
| H17 |  |  |  |  | .778 |  | .636 |
| H18 |  |  |  |  | .585 |  | .631 |
| H19 |  |  |  |  | .707 |  | .596 |
| H20 | .904 |  |  |  |  |  | .831 |
| H21 |  |  | .640 |  |  |  | .565 |
| H22 |  |  |  |  |  |  | .680 |
| H23 |  | .784 |  |  |  |  | .774 |
| H24 | .895 |  |  |  |  |  | .807 |
| H25 |  | .780 |  |  |  |  | .680 |
| H26 |  |  |  |  |  | .545 | .348 |
| H27 |  |  |  |  |  | .612 | .667 |
| H28 |  | .872 |  |  |  |  | .837 |
| H29 |  | .901 |  |  |  |  | .839 |
| H30 |  |  |  |  |  | .664 | .672 |
| H31 | .926 |  |  |  |  |  | .863 |
| H32 |  | .719 |  |  |  |  | .687 |
| Extraction Method: Principal Component Analysis.  Rotation Method: Varimax with Kaiser Normalization. | | | | | | |  |
| a. Rotation converged in 14 iterations. | | | | | | |  |
